# Selflessness is sexy: reported helping behaviour increases desirability of men and women as long-term sexual partners

**DOI:** 10.1186/1471-2148-13-182

**Published:** 2013-09-03

**Authors:** David Moore, Stuart Wigby, Sinead English, Sonny Wong, Tamás Székely, Freya Harrison

**Affiliations:** 1 Centre for Pain Research, University of Bath, Bath, UK; 2 Department of Zoology, University of Oxford, South Parks Road, Oxford, OX1 3PS, UK; 3 Department of Biology & Biochemistry, University of Bath, Claverton Down, Bath, BA2 7AY, UK; 4 Current address: School of Natural Sciences & Psychology, Tom Reilly Building, Byrom Street, Liverpool, L3 3AF, UK; 5 Current address: School of Molecular Medical Sciences, Centre for Biomolecular Sciences, University of Nottingham, University Park, Nottingham, NG7 2RD, UK

**Keywords:** Altruism, Cooperation, Mate choice, Parental care, Sexual selection

## Abstract

**Background:**

Despite its short-term costs, behaviour that appears altruistic can increase an individual’s inclusive fitness by earning direct (selfish) and/or indirect (kin-selected) benefits. An evolved preference for other-regarding or helping behaviour in potential mates has been proposed as an additional mechanism by which these behaviours can yield direct fitness benefits in humans.

**Results:**

We asked 32 heterosexual women and 35 heterosexual men to rate the attractiveness of members of the opposite sex in the presence and the absence of information about helping behaviours. Reports of helping behaviour were associated with a significant increase in the attractiveness of both men and women as potential long-term sexual partners. Altruism also increased the attractiveness of men as potential partners for short-term flings, but to a lesser extent than when the same men were being considered for long-term relationships. Altruism did not affect the attractiveness of women as partners for short-term flings.

**Conclusions:**

Our results unite two important areas of evolutionary theory – social evolution and sexual selection – and extend the list of means by which helping behaviours, which appear at first glance to be costly to the actor, can in fact earn direct fitness benefits. Helping behaviours may be attractive because they signal ‘good genes’ and/or because they are perceived as a signal of likely provision of non-genetic benefits (e.g. parental care). Exactly why helping behaviours in a non-mating context might be attractive to potential mates, and whether they are honest signals of mate quality, remains to be elucidated.

## Background

Acts that appear altruistic – where one individual incurs an immediate cost in order to confer a benefit on another individual – can potentially lead to an increase in the actor’s inclusive fitness that outweighs its short-term costs [1]. Such behaviours can earn direct fitness benefits as a result of mutual benefit, reciprocity, evasion of punishment or enhancement of social status, as well as indirect (kin-selected) fitness benefits (reviewed in [2-4]). An evolved preference for other-regarding behaviour in potential mates has been proposed as an additional mechanism by which helping behaviours can yield direct fitness benefits in humans [5-8]. This hypothesis has not yet received the level of empirical attention paid to other mechanisms by which helping behaviours earn direct fitness benefits.

An evolved preference for helping behaviour in mates is predicted to arise when parents need to cooperate to raise offspring successfully. The incidence of biparental care across the tree of life is sparse, but it is common in birds [9] and canids [10] and is practised by a significant minority of cichlid fishes [11] and primates [12], including humans. (See also [13]). Human babies are born at a very early stage of development and so depend on parental care for survival [14,15]. A correlation between offspring survival and paternal care, especially under subsistence conditions [16], has been cited as one explanation for the prevalence of social monogamy and biparental care in humans ( [17,18], see also [19]). While women pay the larger minimum cost of reproduction due to pregnancy, lactation and a lower reproductive rate, typical paternal investment in our species can be significant [16,20,21]. Thus, both sexes may be predicted to exhibit a significant degree of mate choice based on the likely genetic and/or non-genetic benefits of mating with different individuals [22-24], especially when considering long-term relationships with a higher perceived chance of reproduction [25].

Animals may choose mates based on signals of genetic quality ( [26]; see also [27]. Thus individuals may employ high-cost signals to communicate to potential mates their ability to supply ‘good genes’ and so high-fitness offspring. Therefore it has been hypothesised that helping behaviours may act as costly signals for genetic benefits such as high intelligence or good physical health [6,8,28]. Further, some helping behaviours involve risking one’s own life or physical wellbeing (e.g. jumping into a river to save a drowning person) and there is evidence that risk-taking or ‘heroic’ acts, whether they are apparently helping behaviours or not, function as costly signals of genetic quality in human males [29]. Perhaps more pertinent to understanding the likely links between sexual selection, parental care and mating systems [30] is the hypothesis that one or both sexes may choose mates based on their likely investment in parental care. Indeed, it has been shown empirically that ability and willingness to contribute to childcare may make a man more attractive as a long-term sexual partner [31,32]. Further, a classic study by Buss [33] showed that both men and women value traits such as kindness, sympathy and helpfulness in potential mates, and it might logically be argued that these are facets of what we might call a cooperative personality. Thus, if we think of biparental care as a form of cooperation (offspring being a public good shared by the parents), we might expect that people who display a cooperative or helpful phenotype in non-mating contexts may be perceived as more likely to cooperate in a care context and thus more desirable as sexual partners. According to this hypothesis, non-heroic helping behaviours could function as a signal of ability and/or willingness to supply non-genetic benefits to future mates [34].

Four experimental papers have recently tested the hypothesis that cooperative or helping behaviour is attractive. Farrelly *et al*. [5] report that in dyadic economic games, partners who act more cooperatively are perceived as more attractive; conversely, people preferentially directed cooperative behaviour towards more attractive members of the opposite sex. However, this purely monetary approach makes it difficult to disentangle the effect on attractiveness of cooperativeness from a simpler effect of economic resources, which are known to be valued by women [35-37]. In the second study, Phillips *et al*. [7] used a survey method to report that people find helping behaviours attractive in potential mates and that this preference is more pronounced in females (interestingly, these authors later used a twin study to discover significant genetic effects on this preference: [38]). However, because this study did not compare helping behaviours with a neutral control, it does not allow inferences to be drawn about whether helping behaviour has an absolute positive effect on attractiveness. Third, Barclay [39] presented men and women with vignettes and photographs describing opposite-sex individuals; each participant saw four vignettes, two of which included information on helping activities and two of which presented information on neutral (i.e. not helpful but not selfish) activities. Both men and women rated individuals described as taking part in helping behaviours as more attractive as potential long-term romantic partners; women also preferred helpful males for one-night stands, while this preference was not found in men. Most recently. Farrelly [40] provided evidence that fertility (stage of the menstrual cycle) has little effect on female preferences for helpful males and interpreted this as being consistent with women perceiving helping behaviours as a signal of likely non-genetic benefits [19,32,41]. However, this seems at odds with Barclay's finding that women also found helpful behaviour attractive in a partner for a one-night stand and in these latter two studies each participant rated only eight or four individuals respectively, making for a rather small sample size.

The results of these four studies are intriguing, but it is interesting to note the lack of a simple study that i) tests the hypothesis that a report of helping behaviour makes a given individual more attractive to individuals of the opposite sex, as compared with information that is neutral with regard to attractiveness, and ii) asks participants to rate a large number of opposite-sex individuals in order to reduce potential impact of the ‘baseline’ attractiveness of the individuals being rated. Our objective was to conduct such a study. We employed a within-subjects design to address three specific hypotheses. First, we predicted that heterosexual people would find members of the opposite sex more attractive if they were reported to take part in helping behaviours, as opposed to having a neutral activity (one that contained no information on helping behaviour) reported. Because we focussed on non-heroic, low-risk helping behaviours, our second hypothesis was that the effect of helping behaviour on attractiveness would be stronger when participants rated attractiveness for a long-term relationship as opposed to a short-term fling. This is because considering a long-term relationship is generally taken to lead participants to consider both likely genetic and non-genetic benefits, whereas a partner for a short-term fling is less likely to supply any non-genetic benefits (but see Discussion, below).

## Methods

### Experimental design and participants

**Figure 1:**
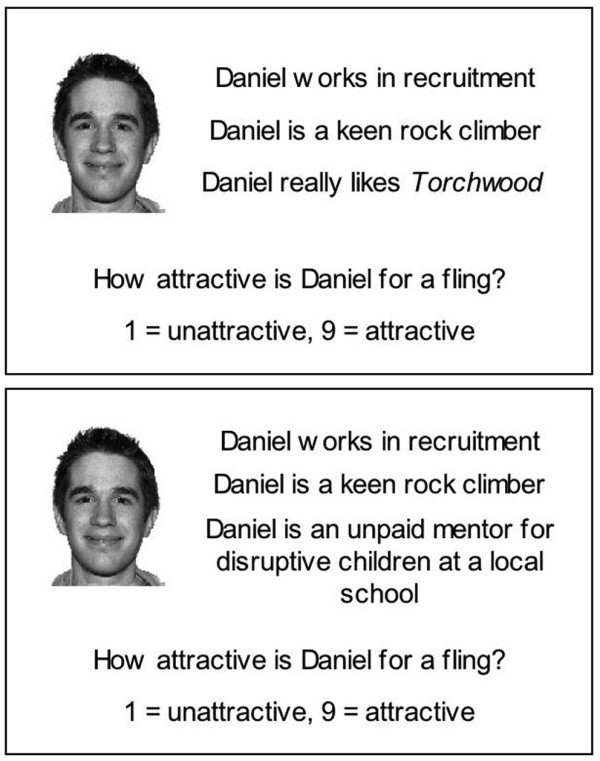
**Stimuli used in the partner rating experiment.** Example of a pair of cards for one target for the short-term relationship condition, showing neutral (top) and ‘altruistic’ (bottom) traits.

We used surveys to construct lists of ‘altruistic’ and neutral traits (see below) and used these to create a series of identity cards, each showing a headshot of an individual (the target) and three statements purporting to be quotes from the target’s friends. We constructed two identity cards for each target in our database (Figure 1). One stated their job, their favourite sport and a characteristic taken from the neutral activity list. The other stated their job, their favourite sport and an activity taken from the ‘altruistic’ list. Cards were created in E-Prime (Psychology Software Tools, Inc. [42]) from lists of jobs, sports, neutral activities and altruistic activities. Photographs were taken from standard databases [43-46] and depicted men or women with happy expressions who appeared to be aged between 18 and 30 years. We excluded job titles that may be seen as suggesting altruistic tendencies, significant ambition, high salary prospects or high intelligence; these last three have been shown to make men more attractive [36].

We recruited 32 heterosexual, childless women with a mean age of 24.1±3.01 years (range: 18–29) and 35 heterosexual, childless men with a mean age of 19.5±1.17 years (range: 18–23). Participants took part in two surveys, rating opposite-sex targets’ attractiveness for short- and long-term relationships using a nine-point Likert scale (1 = very unattractive, 9 = very attractive). In our initial surveys to define lists of ‘altruistic’ behaviours, men collectively saw fewer behaviours as unambiguously altruistic and this meant that we had to define sex-specific lists of altruistic behaviours. Further, this meant that we were able to construct 40 pairs of identity cards depicting men but only 28 pairs of identity cards depicting women. In Survey 1, each participant was shown the neutral or N card for half of the targets and the ‘altruistic’ or A card for the other half. Two weeks later, participants were recalled for a Survey 2. Each was shown the same set of targets as in the first survey, but half of the targets whose A card had been shown in the first survey now had their N card displayed, and half of the targets whose N card had previously been shown now had their A card displayed. Thus each participant experienced four treatment conditions in a 2x2 factorial design (A card shown first *vs*. N card shown first and change *vs*. no change between surveys 1 and 2). No participants dropped out of the study between surveys 1 and 2.

Demographic information was collected at the start of Survey 1. In the case of female participants, we asked them to provide their average menstrual cycle length and the number of days elapsed since the first day of their last period (all female participants reported having regular periods); we also asked if they were using hormonal contraceptives. We collected these data because women have been shown to exhibit greater preference for men displaying ‘good genes’ when they are more likely to conceive and for paternal or partner quality traits when the likelihood of conception is low (reviewed in [19], see also [41,47,48] for examples). Unfortunately, only 13 female participants were not using hormonal contraceptives; further, the participants who were using hormonal contraceptives used a surprising range of methods (pill, subcutaneous implant and vaginal ring). Due to this heterogeneity and the small sample size, we did not test for an effect of contraceptive use on female preferences in the present analysis.

Participants were recruited using posters placed in University departments and Oxford colleges, via departmental email lists and by directly recruiting people attending the Department of Psychology at the University of Bath to take part in other, unconnected experiments. A minority of participants were the result of snowball sampling, where participants recruited their friends. Participants received £2 worth of shopping vouchers in return for taking part in the experiment. We followed the ethical guidelines of the British Psychological Society and the Universities of Bath and Oxford in the design and implementation of this research. Ethical approval was obtained from the University of Bath and from the University of Oxford’s Inter-Divisional Research Ethics Committee (Ref. SSD/CUREC1/10-270). All participants supplied written informed consent.

### Lists of helping behaviours and neutral behaviours

Based on the items used by Phillip’s *et al*. [7] and on discussions with colleagues, we compiled a list of activities that may be seen as ‘altruistic;’ we use this word in its everyday sense as referring to behaviour that confers some immediate cost to the actor and some immediate benefit to a recipient, rather than the strict biological sense, which defines costs and benefits with regard to lifetimes fitness [1,3,4].

We then used an online survey to determine whether these activities were consistently seen as being altruistic: 89 men and 72 women between the ages of 18 and 30 were recruited using university mailing lists and online communities and were asked to rate each activity from “not at all altruistic” to “very altruistic,” using a five-point Likert scale. We retained activities that showed significant skew towards high altruism ratings and a modal rating of 4 or 5; the results of this analysis and the activities used in the main experiment are given in Additional file 1: Table S1. The helping behaviours used were a mix of behaviours involving donation of time and effort to help others (e.g. “she/he does the shopping for her/his elderly neighbour,” “she/he is an unpaid mentor for children at a local school,” “she/he volunteers at a homeless shelter”). As far as possible, we avoided using traits that could be seen as ‘heroic’, as physical risk-taking and bravery has been shown to be an attractive male quality and a potential confounding factor in judging the attractiveness of helping [29]; only one of our helping behaviours (being a volunteer coastguard) could be argued to involve significant personal physical risk. We also avoided using charitable acts that involved donating large amounts of money as a large disposable income is inherently attractive [35-37].

It is very difficult to find activities or preferences that are truly neutral with regard to attractiveness – especially when they must not stand out as obviously fake. For our neutral traits, we compiled a list of preferences for food, music or entertainment consoles that are appropriate for 18-30-year-olds (e.g. “The last film she/he saw was *Duplicity*,” “She/he loves Chinese food,” “She/he enjoys playing with her/his Playstation3”). We again used online surveys to gain some insight into the effect of these traits on attractiveness. 80 men and 91 women between the ages of 18 and 30 were asked whether each activity would affect how attractive they found a member of the opposite sex using a five-point Likert scale ranging from −2 to +2, where 0 indicated “no effect on attractiveness.” Because individual preferences for these relatively unimportant activities are likely to vary widely among people, we hypothesised *a priori* that, if there really is no skew across the whole population, the probability of obtaining significant skew in a random sample of <100 people is relatively high; i.e. 100 becomes a small sample size and a test for skewness is likely to be prone to a high Type I error rate. We therefore reduced the significance level of the test for skew to *alpha *= 0.01 and none of the tests was significant at this level. The list of traits used is given in Additional file 2: Table S2.

### Data analysis

Data were analysed in R version 2.15.2 [49] using linear mixed effect models (lme4 package: [50]). We thought it important to first determine the repeatability of participants’ responses to targets across the two surveys; if participants’ ratings of targets presented with identical information on both occasions (the no-change condition) is not repeatable, then this variability would introduce a significant amount of noise into data collected from the change condition. This could lead to a reduction in power of any test for the effect of adding or removing information about altruism. We calculated a measure of adjusted repeatability using the *rptR* package [51] using linear mixed effect models to account for potential effects of card condition (A or N) and whether ratings were provided for a long- or short-term relationship. By including random factors of participant identity, card identity and participant-card combination, we could estimate repeatability for participants rating specific cards while accounting for variance due to repeated measures on participants and cards. We measured repeatability separately for male and female participants. To analyse the effect of perceived altruism on attractiveness, we tested how changing the condition of the cards from neutral to altruistic, or *vice versa*, affected the attractiveness ratings given to the cards. For cards in which condition changed between trials, we subtracted the score given in the second card viewing from the score given in the first viewing. We then tested whether the condition at the second trial (i.e. neutral or altruistic), and the length of the relationship being considered, affected the difference in attractiveness ratings between the trials. We constructed linear mixed effect models in which trial 2 condition and relationship length were fixed factors; we also included as a fixed factor a quantitative measure of how altruistic the specific trait used on the A card was perceived to be by respondents in our online survey (*z*-transformed skew of ratings provided by respondents of the same sex as the current participants: see Additional file 1: Table S1). Finally, we included interactions between trial 2 condition and relationship length, and between trial 2 condition and *z*-skew. Participant identity and participant-card combination were included as random effects to account for variance due to repeated measures on participants and cards. We tested the significance of fixed factors by conducting likelihood ratio tests on models with and without the fixed factor. The *lsmeans* package [52] was used to obtain least square means and standard errors for each treatment level, and to perform Tukey tests comparing each treatment level. Male and female participant data were analysed separately. We conducted an additional analysis on female participant data where we excluded participants who were over 23 years of age (the maximum age of our male participants). This was to check that any differences in patterns between the sexes were not attributable to older average age of female, compared to male, participants. The mean age of women in this group was 21±1.65 years (range: 18–23) Data and R code are supplied in Additional file 3: Data supplement.

## Results

### Card rating repeatability among male and female participants

Participants gave significantly repeatable ratings of the same card across trials (male participants, *R* = 0.421 [0.390, 0.457]; female participants: *R* = 0.235 [0.205, 0.265]), accounting for potential effects of card condition and relationship length, and for variation among participants and among cards.

### Effect of altruism on card rating in the change condition: male participants rating female cards

**Figure 2:**
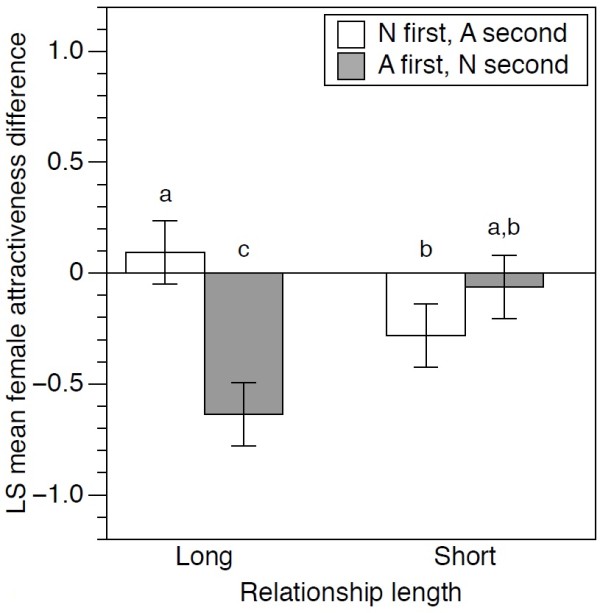
**Altruism significantly increased female attractiveness rating for long-term relationships.** The effect of altruistic *versus* neutral traits on female attractiveness for long and short term relationships. Cards were rated for attractiveness (on a 9-point Likert scale) and where the altruism card was shown in one trial, the neutral was shown in the other. The graphs show the least-square mean (± SE) change in attractiveness between trials (trial 2 value minus trial 1 value) with positive values indicating that the rating was higher in the 2^nd^ trial than in the 1^st^ trial. Bars with different letters above them are significantly different from each other using Tukey corrected multiple comparisons.

We found significant interactions between trial 2 condition and relationship length (χ^2^_1_ = 47.88, p <0.0001), indicating that the influence of altruistic traits, relative to neutral traits, on attractiveness depends on whether long-term or short-term relationships are being considered (Figure 2). Cards with altruistic traits were rated higher than those with neutral traits for long-term relationships, but there was no difference between altruistic and neutral ratings for short-term relationships, and in fact the trend was in the opposite direction (Figure 2). We detected a significant interaction between trial 2 condition and *z*-skew (χ^2^_1_ = 4.865, p =0.0274) showing that, as expected, altruistic traits with higher *z*-skew values were rated as more attractive.

### Effect of altruism on card rating in the change condition: female participants rating male cards

**Figure 3:**
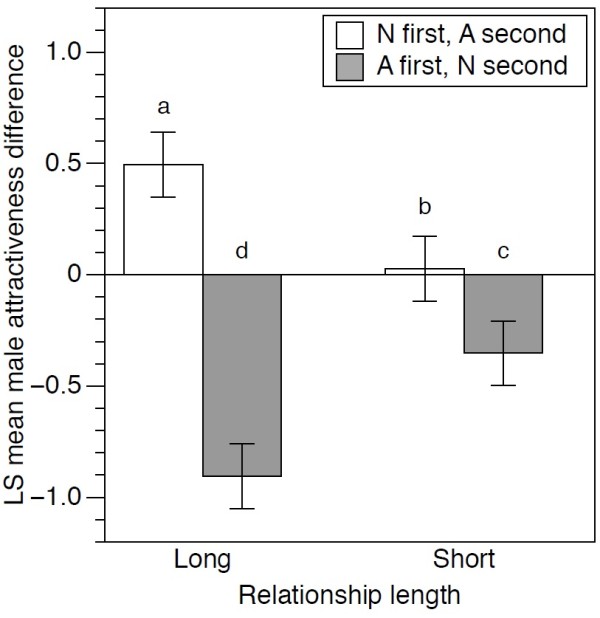
**Altruism significantly increased male attractiveness rating for long-term relationships.** The effect of altruistic *versus* neutral traits on male attractiveness for long and short term relationships. Cards were rated for attractiveness (on a 9-point Likert scale) and where the altruism card was shown in one trial, the neutral was shown in the other. The graphs show the least-square mean (± SE) change in attractiveness between trials (trial 2 value minus trial 1 value) with positive values indicating that the rating was higher in the 2^nd^ trial than in the 1^st^ trial. Bars with different letters above them are significantly different from each other using Tukey corrected multiple comparisons.

We found a significant interaction between trial 2 condition and relationship length (χ^2^_1_ = 55.08, p < 0.0001) which, as for males, shows that the effect of perceived altruism on attractiveness depended on whether long-term or short-term relationships were being considered (Figure 3). However, in contrast to the male participant data, altruistic traits resulted in significantly higher attractiveness ratings, relative to neutral traits, for both long and short-term relationships (Figure 3), thought the effect was smaller for short-term relative to long-term relationships. We detected no significant interaction between trial 2 condition and *z*-skew (χ^2^_1_ = 1.17, p =0.279), suggesting that the strength of altruistic trait did not predict the attractiveness scores given by women participants. In order to verify that differences in response to altruistic traits between the sexes could not be explained by the overall higher average age of female participants, as compared to male participants, we performed an additional analysis on a subset of the data that excluded participants over 23 years old. This revealed a similar pattern to the full dataset (Additional file 4: Figure S1), with a significant interaction between trial 2 condition and relationship length (χ^2^_1_ = 16.52, p <0.0001) but no significant interaction between trial 2 condition and *z*-skew (χ^2^_1_ = 0.293, p =0.588).

The overall negative skew in attractiveness differences suggests that participants, especially men, tended to rate cards higher in trial 1 relative to trial 2.

## Discussion

Our results provide support for our two hypotheses. Participants rated individuals as significantly more attractive as potential long-term partners when they were reported to display helping behaviours. Reported helping behaviour had a smaller effect on male attractiveness, and no significant effect on female attractiveness, when the same individuals were rated as potential partners for a short-term fling. Preferences for traits that signal genetic quality are generally predicted to be increased when short-term, as opposed to long-term, relationships are being considered; however, the absence of such a contrast is not sufficient evidence for a lack of genetic benefits to mating with helpful individuals – especially if the likelihood of conception from a short-term fling is perceived to be low. It is conceivable that the helping behaviours we used could signal genetic benefits, non-genetic benefits or both. Potential non-genetic benefits could result from more helpful individuals investing greater effort into parental care or from helping behaviours cementing an individual’s position in his or her social group, enhancing his or her status [53] or implying that he or she will be the beneficiary of reciprocally helpful acts from other group members [2]: all of these could have positive consequences for his or her mate and family.

Men and women showed qualitatively similar responses to reported helping behaviour when rating targets for a long-term relationship, though helping behaviour had a quantitatively larger effect on women’s rating of men than *vice versa*. It is not possible, however, to test for an effect of participant sex on the enhancement of attractiveness by reported helping behaviour. This is because of a fundamental inability to expose men and women to the same treatments, i.e. heterosexual had to be shown pictures of women and heterosexual women had to be shown pictures of men. Other authors [7] report a stronger response to helping behaviours in women. An unexpected potential effect of sex was revealed in the surveys we used to define lists of ‘altruistic’ helping behaviours: women collectively saw more behaviours as ‘altruistic’ and the lists of behaviours seen as ‘altruistic’ by men and women did not entirely overlap (Additional file 1: Table S1). Whether men and women really do interpret helping behaviours differently would be an intriguing area for future work.

An important caveat to bear in mind when considering our results is that it is difficult to unequivocally identify a perfect control condition with which to compare reports of altruistic behaviour. We chose as our control traits that did not appear to influence attractiveness. Two criticisms of this approach are, first, that it is very hard to find traits that are truly neutral with regard to attractiveness and, second, that any truly neutral traits are not an appropriate control because any vaguely positive trait that is even slightly more informative than the neutral traits could result in increased attractiveness – regardless of what it might signal. In our analysis, helping behaviours that were more often rated as ‘altruistic’ had a greater positive effect on female attractiveness, which suggests that their effect is due to their ‘altruistic’ nature and whatever this might signal. However, we did not find such a relationship in the analysis of male attractiveness. An alternative hypothesis would be to compare attractiveness of targets with reports of helpful and unhelpful behaviours, e.g. “he agreed to donate bone marrow when asked” *versus* “he did not agree to donate bone marrow when asked.” However, in this case the criticism could be levelled that any difference in attractiveness is due to a bias against any form of anti-social behaviour or non-complaisance, rather than a bias towards ‘altruism’ or helping. This is a subtler problem than it might first appear and would benefit from a more thorough investigation.

Genetic and comparative physiological studies are consistent with the human mating system as being generally monogamous with occasional harem polygyny, low extra-pair paternity and minimal differences in parental investment (e.g. [54]). It is therefore plausible that (some) people honestly signal their potential or intended contribution to care [34,55], but this would benefit from future empirical study. Further, it is important to acknowledge that there is continued debate about the relationship between the idealised mate preferences expressed in a controlled and contrived laboratory setting, and the choices actually made in a real-world mating market, with restrictions imposed by an individual’s own mate value and limitations on the number and range of potential mates encountered [19,56]. Phillips *et al*. [7] report a positive correlation between the strength of their participants’ preferences for helping behaviours and the self-reported levels of helping behaviour displayed by their real-world partners. Similarly, DeBruine *et al.*[57] found a positive correlation between the strength of women’s preferences for male facial masculinity and the facial masculinity of their real-world partners. Thus experiments such as ours can provide some insight into the likely existence and direction of sexual selection.

## Conclusions

It is clear that a variety of selective forces combine to increase the inclusive fitness of individuals that display helping behaviours, and thus maintain apparently altruistic or cooperative strategies in populations [2,3]. Furthermore, it is increasingly clear that mate choice and parental interactions should be considered within the more general framework of social evolution [20,30]. Our study supports and verifies previous empirical work on sexual selection for prosocial behaviour: in conclusion, this work adds new weight to the suggestion that we should add sexual selection to the list of mechanisms likely to explain the maintenance of prosocial behaviour in our species.

## Competing interests

The authors declared that they have no competing interests.

## Authors’ contributions

FH conceived the study, recruited participants, collected data, analysed survey data and drafted the manuscript. DM developed the experimental design, programmed it in E-Prime, recruited participants, collected data and contributed to manuscript preparation. SW & SE analysed attractiveness rating data and contributed to manuscript preparation. SW recruited participants and collected data. TS advised on study implementation and contributed to manuscript preparation. All authors read and approved the final manuscript.

## Supplementary Material

Additional file 1: Table S1 Results of online surveys to define ‘altruistic’ behaviours. Items originally proposed as potentially altruistic traits and how they were scored by respondents to an online survey of a) 72 women with a mean age of 24.0±3.25 years, and b) 89 men with a mean age of 23.8±4.08 years. Survey respondents were asked the following: “In your opinion, are the following activities altruistic? Please rate each activity from 1–5, where 1 = not at all altruistic and 5 = very altruistic.” Responses to each item were analysed for skewness and items which did not show significant negative skew (*p* ≥ 0.05) were dropped from the list and not used inteh experiment. Items which had a modal rating of <4 were also dropped (no item had a mode of 5). Items dropped from the list are highlighted in grey. This left 20 items which we classed as altruistic for females and 12 for males. Internal consistency was assessed using Cronbach’s alpha. This measures the extent to which a set of variables measures a single, unidimensional underlying construct. Our values of alpha were high (0.99 for 20 items seen as altruistic by females and 0.94 for 12 items seen as altruistic by males), suggesting that the responses to different activities were consistent. Click here for file

Additional file 2: Table S2 Results of online surveys to define ‘neutral’ behaviours. 80 men and 91 women aged 18–30 were asked whether each activity would affect how attractive they found a member of the opposite sex using a five-point Likert scale ranging from −2 to +2, where 0 indicated “no effect on attractiveness.” We retained traits which did not produce significant skew in responses (alpha=0.01) and these are listed below. Click here for file

Additional file 3 Data supplement. Click here for file

Additional file 4: Figure S1. The effect of altruistic *versus* neutral traits on male attractiveness for long and short-term relationships, using only data collected from female participants aged ≤23 years. Cards were rated for attractiveness (on a 9-point Likert scale) and where the altruism card was shown in one trial, the neutral was shown in the other. The graphs show the least-square mean (± SE) change in attractiveness between trials (trial 2 value minus trial 1 value) with positive values indicating that the rating was higher in the 2^nd^ trial than in the 1^st^ trial. Bars with different letters above them are significantly different from each other using Tukey corrected multiple comparisons. Click here for file
