# Supplementary material for: Selflessness is sexy: reported helping behaviour increases desirability of men and women as long-term sexual partners
Source: BMC Evol Biol. 2013 Sep 3;13:182. doi: 10.1186/1471-2148-13-182 (PMC3851331; doi:10.1186/1471-2148-13-182)
Supplement: Additional file 1: Table S1 — Results of online surveys to define ‘altruistic’ behaviours. Items originally proposed as potentially altruistic traits and how they were scored by respondents to an online survey of a) 72 women with a mean age of 24.0±3.25 years, and b) 89 men with a mean age of 23.8±4.08 years. Survey respondents were asked the following: “In your opinion, are the following activities altruistic? Please rate each activity from 1–5, where 1 = not at all altruistic and 5 = very altruistic.” Responses to each item were analysed for skewness and items which did not show significant negative skew (p ≥ 0.05) were dropped from the list and not used inteh experiment. Items which had a modal rating of <4 were also dropped (no item had a mode of 5). Items dropped from the list are highlighted in grey. This left 20 items which we classed as altruistic for females and 12 for males. Internal consistency was assessed using Cronbach’s alpha. This measures the extent to which a set of variables measures a single, unidimensional underlying construct. Our values of alpha were high (0.99 for 20 items seen as altruistic by females and 0.94 for 12 items seen as altruistic by males), suggesting that the responses to different activities were consistent. [file 1471-2148-13-182-S1.pdf]

Table S1. Results of online surveys to define 'altruistic' behaviours

Items originally proposed as potentially altruistic traits and how they were scored by respondents to an online survey of a) 72 women with a mean age of 24.0±3.25 years, and b) 89 men with a mean age of 23.8±4.08 years. Survey respondents were asked the following: “In your opinion, are the following activities altruistic? Please rate each activity from 1-5, where 1 = not at all altruistic and 5 = very altruistic.” Responses to each item were analysed for skewness and items which did not show significant negative skew ($p \geq 0.05$) were dropped from the list and not used inteh experiment. Items which had a modal rating of <4 were also dropped (no item had a mode of 5). Items dropped from the list are highlighted in grey. This left 20 items which we classed as altruistic for females and 12 for males. Internal consistency was assessed using Cronbach’s alpha. This measures the extent to which a set of variables measures a single, unidimensional underlying construct. Our values of alpha were high (0.99 for 20 items seen as altruistic by females and 0.94 for 12 items seen as altruistic by males), suggesting that the responses to different activities were consistent

Item	a) Female responses N	Mean	Mode	Zskew Statistic	1-tailed *p*	b) Male responses N	Mean	Mode	Zskew Statistic	1-tailed *p*
Sponsoring a Ugandan child to attend school	72	3.85	4	-1.95	0.025	88	3.68	5	-2.59	0.005
Organising a litter pick in a local park	71	3.70	4	-3.30	0.001	89	3.35	3.5	-0.78	0.218
Volunteering to help local school children with their reading	72	3.67	4	-3.78	<0.001	89	3.37	4	-0.34	0.367
Providing respite care for a sick relative*	71	4.07	4	-3.32	<0.001	88	3.61	4	-2.11	0.018
Regularly looking after the children of a relative with multiple sclerosis*	71	4.17	4	-4.25	<0.001	89	3.52	4	-2.81	0.002
Volunteering at a homeless shelter	72	4.19	4	-3.11	0.001	88	3.56	4	-2.64	0.004
Running a marathon to raise money for charity	72	3.28	3	-0.84	0.200	89	3.85	4	-3.65	0.000
Regularly donating blood	71	3.93	4	-1.68	0.047	87	3.09	3	-0.43	0.334
Helping to maintain a nature reserve	71	3.52	4	-2.17	0.015	89	3.65	4	-1.78	0.037
Doing the shopping for an elderly neighbour	72	3.99	4	-4.41	<0.001	89	3.34	4	-0.89	0.187
Recording a 'talking newspaper' serivce for blind and partially-sighted people	70	4.06	4	-3.40	0.000	89	3.73	5	-3.00	0.001
Volunteering to teach your trade to mentally disabled people to help them find employment	72	4.24	4	-2.21	0.014	89	3.73	5	-2.84	0.002
Being a police community support officer	72	3.42	4	-1.66	0.048	89	3.84	3.5	-3.42	0.000
Being a volunteer coastguard	71	3.93	4	-2.91	0.002	89	3.02	4	-0.17	0.432
Volunteering your web design skills to set up a website for a charity	72	3.67	4	-1.42	0.078	88	3.45	3	-1.66	0.048
Being an unpaid mentor for disruptive children at a local school	72	4.24	4	-2.92	0.002	89	3.34	5	-0.99	0.160
Being a volunteer dog walker for an RSPCA animal shelter	71	3.35	3	0.12	0.549	88	3.77	3	-2.68	0.004
Helping to run a local guide or scout group	72	3.43	4	-1.99	0.023	89	3.21	3	-0.27	0.394
Volunteering to help recent immigrants to your country to learn the native language	71	3.83	4	-3.17	0.001	89	3.22	4	-1.01	0.156
Spending a gap year volunteering with a conservation charity in your home country	72	3.39	4	-3.05	0.001	89	3.55	3	-2.20	0.014
Spending a gap year volunteering with a conservation charity abroad	72	3.06	3	-0.94	0.175	89	3.35	3	-1.23	0.109
Spending a gap year teaching in a school in a developing country	72	3.26	4	-1.30	0.097	88	3.07	5	-0.65	0.257
Volunteering for one afternoon a week in a charity shop	72	3.49	4	-0.84	0.200	89	3.48	4	-2.16	0.015
Volunteering at a 'homework club' for school children	72	3.65	4	-3.11	0.001	89	3.35	4	-1.41	0.079
Organising a charity raffle to buy medical equipment for a local hospital	72	3.76	4	-2.46	0.007	89	3.61	4	-2.06	0.020
Volunteering to take part in clinical trials of a new vaccine	72	3.10	3	-0.08	0.534	89	3.54	3	-2.31	0.010
Paying for a niece or nephew to attend university	72	3.78	4	-1.91	0.028	89	2.93	3	0.38	0.648
Donating bone marrow	na	na	na	na	na	87	4.13	5	-5.14	0.000
Volunteering for one afternoon a week at a local hospice	na	na	na	na	na	89	3.72	4	-2.55	0.005
Fasting for twenty-four hours to raise money for charity	na	na	na	na	na	89	3.07	4	-0.20	0.421

Due to the paucity of items identified by male respondents as altruistic, we felt justified in duplicating the two items marked * for male participants by using two specific illness in easch case. Thus "a sick relative" became either "a relative with Alzheimer's disease" and "a relative with cancer." "A relative with multiple sclerosis" was duplicated to "an aunt who is paralysed."
