# Supplementary material for: Selflessness is sexy: reported helping behaviour increases desirability of men and women as long-term sexual partners
Source: BMC Evol Biol. 2013 Sep 3;13:182. doi: 10.1186/1471-2148-13-182 (PMC3851331; doi:10.1186/1471-2148-13-182)
Supplement: Additional file 2: Table S2 — Results of online surveys to define ‘neutral’ behaviours. 80 men and 91 women aged 18–30 were asked whether each activity would affect how attractive they found a member of the opposite sex using a five-point Likert scale ranging from −2 to +2, where 0 indicated “no effect on attractiveness.” We retained traits which did not produce significant skew in responses (alpha=0.01) and these are listed below. [file 1471-2148-13-182-S2.pdf]

Table S2. Results of online surveys to define 'neutral' behaviours

80 men and 91 women aged 18-30 were asked whether each activity would affect how attractive they found a member of the opposite sex using a five-point Likert scale ranging from -2 to +2, where 0 indicated “no effect on attractiveness.” We retained traits which did not produce significant skew in responses (alpha=0.01) and these are listed below.

Items used for female participants	Items used for male participants
He enjoys playing with his PS3	Her favourite TV programme is the Simpsons
He likes dancing to house music	She likes eating Thai food
He likes his Xbox	She likes Indian food
He likes Indian food	She likes listening to bhangra
He likes listening to bhangra	She likes listening to R&B
He likes listening to R&B	She loves her Kindle
He listens to a lot of rock music	She loves watching NCIS
He loves Chinese food	She never misses an episode of the Flight of the Conchords
He loves watching NCIS	She really likes Torchwood
He never misses an episode of the Flight of the Conchords	She's a fan of Thai food
He really likes Torchwood	The last film she watched was District 9
He's a fan of Thai food	The last film she watched was Duplicity
He's a massive fan of Heroes	The last film she watched was Slumdog Millionnaire
He's really into playing on his Wii	The last film she watched was Surrogates
His favourite food is Italian	
His favourite TV programme is the Simpsons	
The last film he saw was Batman Forever	
The last film he saw was Gran Torino	
The last film he watched was Duplicity	
The last film he watched was Slumdog Millionnaire	
