# Supplementary figures and images for: Selflessness is sexy: reported helping behaviour increases desirability of men and women as long-term sexual partners
Source: BMC Evol Biol. 2013 Sep 3;13:182. doi: 10.1186/1471-2148-13-182 (PMC3851331; doi:10.1186/1471-2148-13-182)

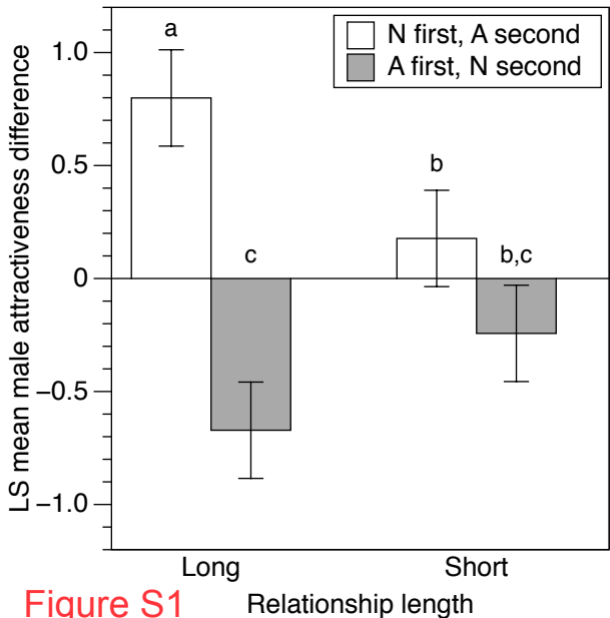

Supplement: Additional file 4: Figure S1. — The effect of altruistic versus neutral traits on male attractiveness for long and short-term relationships, using only data collected from female participants aged ≤23 years. Cards were rated for attractiveness (on a 9-point Likert scale) and where the altruism card was shown in one trial, the neutral was shown in the other. The graphs show the least-square mean (± SE) change in attractiveness between trials (trial 2 value minus trial 1 value) with positive values indicating that the rating was higher in the 2nd trial than in the 1st trial. Bars with different letters above them are significantly different from each other using Tukey corrected multiple comparisons. [file 1471-2148-13-182-S4.pdf]
